# Impaired Recognition of Facially Expressed Emotions in Different Groups of Patients with Sleep Disorders

**DOI:** 10.1371/journal.pone.0152754

**Published:** 2016-04-13

**Authors:** Tatjana Crönlein, Berthold Langguth, Peter Eichhammer, Volker Busch

**Affiliations:** Department of Psychiatry and Psychotherapy, University of Regensburg, Regensburg, Germany; University of Tuebingen Medical School, GERMANY

## Abstract

**Introduction:**

Recently it has been shown that acute sleep loss has a direct impact on emotional processing in healthy individuals. Here we studied the effect of chronically disturbed sleep on emotional processing by investigating two samples of patients with sleep disorders.

**Methods:**

25 patients with psychophysiologic insomnia (23 women and 2 men, mean age: 51.6 SD; 10.9 years), 19 patients with sleep apnea syndrome (4 women and 15 men, mean age: 51.9; SD 11.1) and a control sample of 24 subjects with normal sleep (15women and 9 men, mean age 45.3; SD 8.8) completed a Facial Expressed Emotion Labelling (FEEL) task, requiring participants to categorize and rate the intensity of six emotional expression categories: anger, anxiety, fear, happiness, disgust and sadness. Differences in FEEL score and its subscales among the three samples were analysed using ANOVA with gender as a covariate.

**Results:**

Both patients with psychophysiologic insomnia and patients with sleep apnea showed significantly lower performance in the FEEL test as compared to the control group. Differences were seen in the scales happiness and sadness. Patient groups did not differ from each other.

**Conclusion:**

By demonstrating that previously known effects of acute sleep deprivation on emotional processing can be extended to persons experiencing chronically disturbed sleep, our data contribute to a deeper understanding of the relationship between sleep loss and emotions.

## Introduction

Deficits in emotion processing are a common symptom in many mental disorders [1]. Emotional processing and sleep are closely interwoven, building a complex interaction which is still not completely understood [2]. A brain region of interest is the prefrontal cortex, which seems to be involved in the interaction of emotion and sleep [3]. A recently published review postulates that an amygdala-hippocampus-medial prefrontal cortex network is involved in emotional processing and fear memory during Rapid-Eye-Movement Sleep [4]. This strengthens older studies emphasizing the role of REM sleep not only in emotional regulation but also in emotional responsiveness [5].

There is evidence based on neuroimaging that regions such as the amygdala and hippocampus are active during REM sleep [6]. Furthermore amygdala-hippocampal networks are proposed to be involved in attenuation of experienced affections and decreasing its emotional intensity[7]. It is known that depression is associated with disturbed sleep and alternation in REM-Sleep [8]. There is data that underscore the hypothesis that untreated insomnia may result in depression [9, 10].

However, the role of sleep in regulating emotional processes becomes especially evident in data from sleep deprivation studies. Based on functional imaging, it could be shown that one night of sleep deprivation in normal subjects was associated with an increased amygdale reaction to negative stimuli (pictures), most likely due to a decreased functional connectivity between the amygdala and the prefrontal cortex [11]. On a behavioural and neurophysiological level, the impact of acute sleep loss on emotional processing has been shown in several studies: By using pupillography, an increased reaction to negative emotional pictures after one night of sleep deprivation in comparison to non-sleep deprived persons was seen [12]. In another study one night of sleep deprivation as compared to undisturbed sleep resulted in a more negative judgement of neutral pictures [13]. Moreover, sleep-deprived persons react more negatively toward disruptive events [14, 15]. Several findings on experimental sleep loss and emotional reactivity which were reviewed systematically, underline the hypothesis that sleep is important in order to maintain adaptive emotional regulation and reactivity [2].

Recognition of facial expression (FER) is a frequently-used paradigm [16] for measuring emotional processing. Two studies demonstrated an impairment in the recognition of facial expression after one night of sleep deprivation [17, 18], which was normalized after one night of recovery sleep [17].

In summary, there is strong evidence that disturbed sleep has a direct impact on emotional processing, leading to a higher sensitivity to negative emotional stimuli. This leads to the question whether persons suffering from sleep disorders are also affected by these processes. Considering the high prevalence of sleep disorders among the population [19, 20] the potential impact of disturbed sleep on emotional regulation would be of highest socio- and health-economic interest.

In a recent pilot study Kyle et al. [21] investigated emotional perception in insomnia patients compared to normal controls and found that insomnia patients rated facial expressions of sadness and fear as emotionally less intense. These findings suggest that alterations of emotional processing are not only present after acute sleep deprivation, but also in patients with chronic insomnia. However, it is still unclear whether the observed alterations of emotional processing in patients with chronically disturbed sleep are specific to psychophysiological insomnia with its particular psychologic and physiologic symptoms [22] or whether similar effects are also observed in other sleep disorders. To address this question we investigated emotional reactions in patients with insomnia and also in a further group of patients with sleep disorders, namely sleep apnea. Sleep apnea is characterized by sleep fragmentation and therefore these patients are a good model for the impact of disturbed sleep on emotions and cognitions. In contrast to patients with insomnia, disturbed sleep here is caused not by central-nervous processes such as hyperarousal, but rather by respiratory events, namely apneas during sleep[23]. To the best of our knowledge, this is the first study to investigate emotional reactivity in this group of patients.

## Materials and Methods

The study was approved from the local ethic comitee at the University of Regensburg (08/102). All participants gave written informed consent for participation in the study.

### Sample

Insomnia patients were recruited in the sleep laboratory of the sleep clinic in Regensburg, Germany. All patients were admitted to our clinic in order to participate in a standardized program for cognitive behaviour therapy for insomnia [24]. Diagnosis was validated according to the ICSD-2 criteria [20] and a polysomnography to exclude the presence of sleep apnea or periodic leg movements in sleep. Inclusion criteria were: At least one month of insomnia; evidence of conditioned sleep difficulty and/or heightened arousal in bed; insomnia was not due to any other current primary sleep, medical or psychiatric disorder. Affective disorders, especially depression, were excluded in a structured interview conducted by an experienced psychiatrist. Any severe neurological or other medical condition led to the exclusion of the patients. 25 insomnia patients with a mean age of 51.6 years (SD: 10.9), 23 females, were tested.

The sleep apnea (SAS) group consisted of 19 patients (4 females and 15 men). Mean age was 51.9 years (SD: 11.1). Inclusion criteria for the SAS groups were an apnea-hypopnea-index > 10/h, disturbed objective sleep and impaired daytime well-being. All patients underwent one night of polysomnography in our sleep clinic.

Normal controls were recruited by a newspaper advertisement. All controls had to fill out a sleep questionnaire (Pittsburgh Sleep Quality Index, PSQI) and were interviewed by a psychiatrist in order to exclude major psychiatric disorders like depression. PSQI [25] is the standard for measuring sleep quality in sleep medicine. A score higher than six is considered to be pathological. 9 men and 15 women were recruited for the control group. The mean age (45.3 ys, SD: 8.8) was slightly lower than the mean age of the patient samples, but the difference did not reach significance (ANOVA F_(2,65)_ = 2.997, p = .057). Moreover, we did not find any relationship between the FEEL scores and age in the control group (Spearman rho = .057; p = .792).

All participants gave written informed consent for participation in the study, which was approved by the local ethical committee.

### Measurements

#### Test

The Facial Expressed Emotion Labelling (FEEL) test is a reliable and valid tool [26] for measuring the ability to recognize facially expressed emotions. Pictures of 6 different emotions (anger, fear, sadness, happiness, surprise and disgust), are presented on screen for 300 ms. Expressions were taken from the JACFEE (Japanese and Caucasian Facial Expressions of Emotion; Matsumoto & Ekman, 1988) series. Procedure of the test: Instructions for the test had to be read by the patients on the monitor. The practice trial was as follows: a neutral face was shown for 1.5s followed by 1s blank screen and the facial test expression. Six words (Anger, Anxiety, Surprise, Sadness, Happiness and Disgust) were presented with the face and one had to be chosen by clicking on it (forced-choice response format). Response time was limited to 10 sec. This was followed by a written feedback about the correctness of the answer. Every one of the six basic emotion expressions was presented in this learning trial. Each participant had then the possibility to ask questions about the procedure. During the actual testing, the procedure was the same except for the feedback. Each emotion was shown 7 times in different faces (half Caucasian and European, half females and males) in randomized order. In total, 42 pictures were shown (seven examples of all six emotions used), resulting in a maximum score of seven points in each emotional category and a maximum total score of the FEEL-Test of 42 points.

#### Polysomnography

All subjects were evaluated for one night in the sleep laboratory. Full cardio-respiratory polysomnographic recordings were performed for 8 hours including electroencephalogram, electrooculogram, electromyogram of the chin muscle, tibialis anterior muscles bilaterally, recording of nasal and abdominal respiration and electrocardiogram and scored according to the manual of the American Academy of Sleep Medicine. Sleep stages and respiratory events were classified by a trained staff member according to standardized guidelines. The following sleep parameters were recorded in order to outline the sleep quality of our samples: Sleep Onset Latency (SOL), Total sleep time (TST), Sleep efficiency (SE); Wake time after sleep onset (WASO); Sleep period time (SPT), Time in bed (TIB), N 1 (% of SPT), N 2 (% of SPT), N 3 and REM sleep (% of SPT). The results are displayed in Table 1.

**Table 1:** Polysomnographic data from one night of insomnia patients (INS) and patients with sleep apnea (SAS). Means and standard deviations.

	Insomnia	Sleep apnea syndrome
N	25	19
Sleep latency to sleep onset (min.)	26.7 ± 19.1	23.0 ± 23.0
Total sleep time (min.)	303.5 ± 70.2	356.4 ± 93.7
Time in bed (min.)	444.1 ± 33.5	464.8 ± 38.8
Sleep efficiency	67.4% ± 13.1	75.8% ± 17.1
Sleep period time (min.)	410.4 ± 43.7	437.0 ± 86.4
Wake after sleep onset (min.)	109.4 ± 43.6	91.3 ± 60.0
Sleep stage 1 (% of SPT)	10.6 ± 5.5	16.0 ± 5.1
Sleep stage 2 (% of SPT)	38.0 ± 11.4	59.1 ± 73.5
Delta sleep (% of SPT)	11.2 ± 7.1	7.8 ± 8.1
Wake (% of SPT)	27.2 ± 12.9	19.4 ± 11.1
REM (% of SPT)	12.8 ± 5.5	13.5 ± 6.8

### Analysis

FEEL total score and FEEL subscores (according to the emotions anger, fear, sadness, happiness, surprise and disgust) were compared across the three samples (Insomnia, sleep apnea and normal) by separate ANOVAs with gender included as a covariable. Given the difference in the gender distribution of the two samples, analysis was also performed without gender as covariate.

The level of significance was set to .05. In case of significant differences, Bonferroni posthoc tests were performed with t-tests. Sleep duration (TST) and wake time after sleep onset (WASO) as parameters for sleep quality (measured by polysomnography) were correlated with the FEEL score using the spearman correlation coefficient. We considered only those two parameters because we had hypothesized that sleep duration (measured by TST) and disturbed sleep (measured by WASO) were associated with the FEEL score.

Analysis was calculated using SPSS 22.0.

## Results and Discussion

Both groups showed low sleep efficiency values (67% for insomnia patients and 76% for sleep apnea patients) mainly due to increased wake time after sleep onset (109 min. for insomnia patients and 91 min. in sleep apnea patients) during the night (Table 1). No correlation between sleep duration and FEEL score nor between wake time after sleep onset and FEEL score was found for any of the patients.

FEEL scores were 35.4 ± 3.6 for the control group, 31.9 ± 4.9 for Insomnia patients and 31.8 ± 4.1 for sleep apnea patients. FEEL-scores differed across groups (ANOVA: F_(2,64)_ = 5.158 p = .008), whereas gender did not have a significant effect on the FEEL score (ANOVA: F_(1, 64)_ = 0.21 p = .884). Repeating the analysis without gender as a covariate did not change the results (F _(2,65) =_ 5.249; p = .008).

In the post hoc test (Bonferroni) insomnia patients (p = .019) and sleep apnea patients (p = .024) differed significantly from the control group, but not from each other.

**Fig 1:**
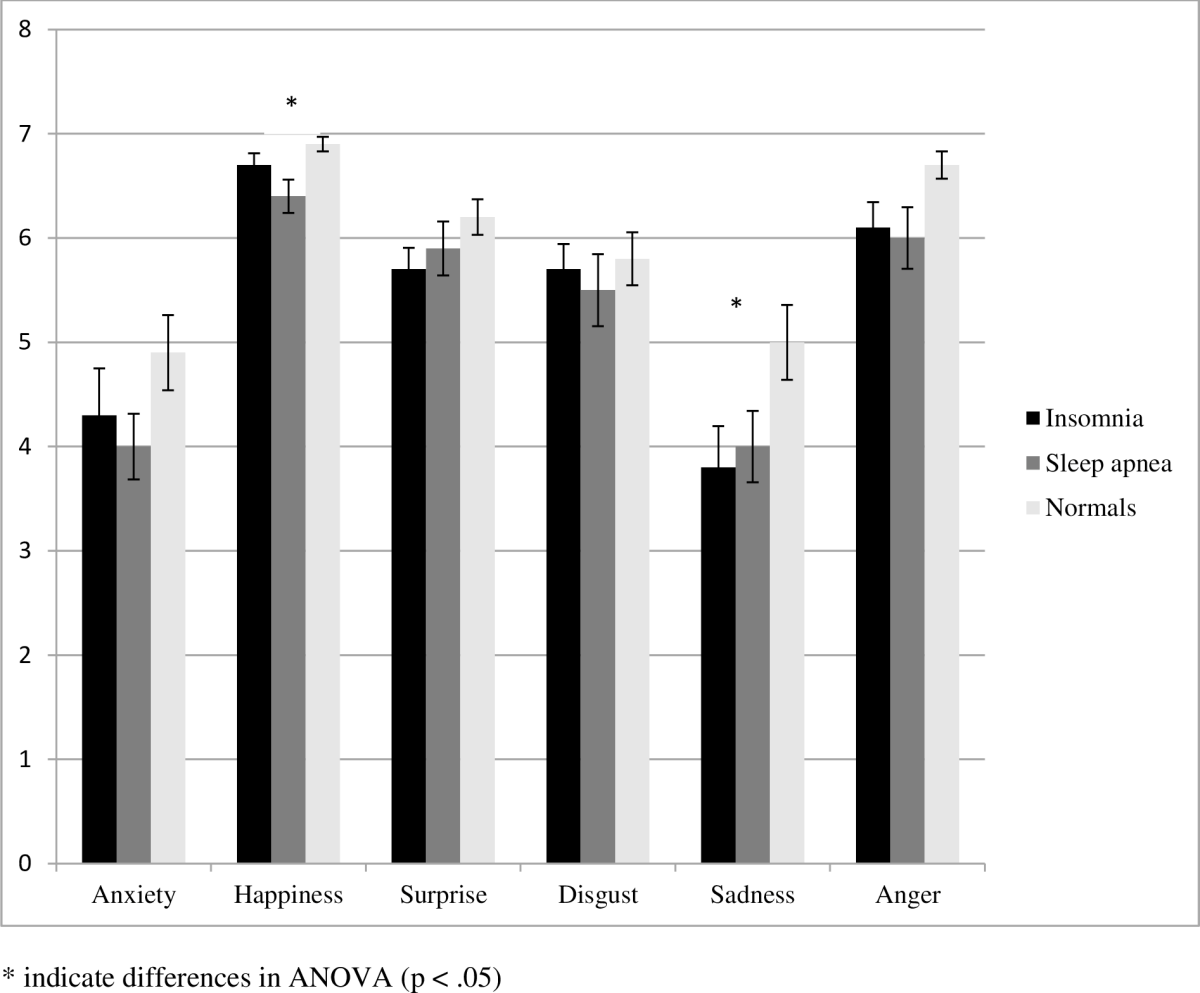
Scores (means and standard errors of the mean) in subscales of the FEEL Test measured in 25 insomnia patients, 19 patients with sleep apnea and 24 controls. * indicate differences in ANOVA (p < .05)

Among the ANOVAs of the subscales the analyses for happiness (F_(2,64)_ = 4.086; p = .021) and sadness (F_(2,64)_: = 3.275; p = .044) revealed significant results (Fig 1). Calculating the analysis without gender as a covariate did not change the results, neither for the scales happiness (happiness: F_(2,65)_: = 3.800; p = .028) and sadness (F_(2,65)_: = 3.213; p = .047) nor for the other scales where no significant differences had been found (Anxiety F_(2,65)_: = 1.398; p = .254; Surprise F_(2,65)_: = 1.485; p = .234; Disgust F_(2,65)_ = .315; p = .731; and Anger F_(2,65)_ = 2.621; p = .080). There was no gender effect in any of the ANOVAS of these two subscales or the other subscales.

In comparison to healthy controls, patients with sleep apnea showed lower scores in the recognition of Happiness (p = .023).

To the best of our knowledge this is the first study demonstrating impaired accuracy of recognition of facially expressed emotions in different samples of patients with sleep disorders. The insomnia patients as well as patients with sleep apnea performed worse in the identification of basic emotions in facial expressions compared to normal subjects (Table 2). There were no differences in the FEEL scores between insomnia patients and patients with sleep apnea. Our data indicate that facially expressed emotion recognition is not only affected by acute sleep deprivation but is also evident in persons with sleep disorders.

**Table 2:** Percentage of false choices in relation to presented faces with specific emotion of insomnia patients, sleep apnea patients and healthy controls in every emotion of the FEEL test.

	Anxiety	Happiness	Surprise	Disgust	Sadness	Anger
24 Healthy persons	29.8%	1.8%	11.3%	17.3%	28.0%	4.8%
25 Insomnia patients	29.9%	4.6%	18.3%	18.9%	45.1%	13.1%
19 Sleep apnea patients	42.9%	8.3%	15.0%	21.8%	42.6%	14.3%

Percentage of false choices refers to the number of presented faces with specific emotion.

By demonstrating impaired facial emotion recognition in chronically sleep-deprived patients, our results expand upon the findings from a recent study in insomnia patients [21]. Similar to the study mentioned, we could not detect a correlation between polysomnographic findings and emotion categorization performance. Given the variability of both measurements, larger sample sizes would probably be required to detect such a direct relationship. In addition to the study of Kylie et al. we also investigated a patient group with sleep apnea syndrome. In contrast to insomnia, where the sleep disturbance is related to various psychological factors, the disturbed sleep in sleep apnea syndrome has a clearly defined organic cause. Our finding that both groups had very similar impairments in recognition of emotional states suggests disturbed sleep as the common cause.

We are aware of the fact that no final conclusions about causal relationships between chronic sleep impairment and impaired emotional processing can be inferred from our cross-sectional data. Longitudinal interventional studies would be needed to investigate potential causal relationships. Further limitations of our study include the variability in the FEEL scores within the groups, and the fact that the three groups were not gender-matched. The unequal gender distribution in the different samples is a weakness of the study. It is caused by variable prevalence rates of the investigated sleep disorders in males and females. Insomnia predominantly affects women, whereas sleep apnea is more prevalent in men. We addressed the latter issue by including gender as a covariate in the statistical analysis.

Age is also an important issue regarding sleep deprivation and emotional regulation and although in our control group age did not have an effect on performance in recognition of facial expressed emotions this issue should be observed in further studies. It is possible that consequences of sleep deprivation can better be compensated at a younger age. We did not focus on this issue in our study because most patients with insomnia and sleep apnea are middle-aged and older [20]. Also we cannot exclude that our results are confounded by specific personality traits. It is known, that insomnia patients show more sleep related worries, that prevent them from falling asleep and it is possible that this higher level of concerns may also affect their concentration on specific tasks. However, we found comparable emotion detection deficits in patients with insomnia as well as in patients with sleep apnea syndrome, thereby considering our observed effects primarily driven by personality traits. Nevertheless future studies should additionally assess personality traits to quantify their influence on the interaction between sleep deprivation and emotion processing.

The finding that acute sleep deprivation and chronic sleep disorders have similar effects on emotion processing parallels findings from neuroimaging studies, which also provide a hint for the underlying neuronal mechanisms. There is evidence that acute sleep loss is associated with down regulatory processes in prefrontal cortex [11]. Hypoactivation of prefrontal cortex has also been described in insomnia patients [27]. Similarly, abnormal amygdala activity and connectivity has been described both after acute sleep deprivation and also in insomnia patients [28, 29].

We are aware that we cannot conclude concisely from our data whether sleep deprivation has a specific effect on emotion processing or whether the observed deficit is just one aspect of a global cognitive and emotional impairment after sleep deprivation. It is well known from the literature that sleep deprivation affects not only psychomotor and cognitive functions, but also working memory and vigilant and executive attention [30]. Moreover, in a recently published meta-analysis reduced brain activation across different tasks after sleep deprivation was reported [31]. Based on these findings, future studies should assess the effect of sleep deprivation on emotional, attentional and cognitive function, in order to differentiate between more global and more specific effects.

By demonstrating that chronically disturbed sleep impairs emotion recognition, these results open a new perspective for understanding affective disturbances in persons with disturbed sleep. In line with this hypothesis is the clinical observation that many insomnia patients report feeling completely different after a good night of sleep and many patients perceive their depressive mood as a direct consequence of their bad sleep.

Further support for the hypothesis of a direct relationship between sleep and emotional processing comes from studies in depressive patients where treatment with the sleep improving melatonergic agent agomelatine results in a more pronounced improvement of emotional reactivity as compared to other antidepressants [32, 33]. Further studies measuring the acute effect of sleep on emotional processing in patients with sleep disorders will be needed to further elucidate a potential causal relationship between sleep at night and emotional processing during the day.

The existence of disturbed emotional regulation or emotional distress in patients with sleep apnea has attracted little scientific interest up to now. However, mood alterations have been observed in sleep apnea patients [34] with the prevalence rates of depression between 17% [35] and 22% [36]. Consistent with our findings of sleep disturbance as the relevant factor for disturbed emotional processing, depressive symptoms in SAS patients are related to non-restorative sleep [37]. Successful treatment of the sleep apnea syndrome also improves residual depressive symptoms [38]. Whether the impairment in emotion processing is merely due to psychological distress mediated by tiredness or whether chronic sleep disturbance has a direct impact on emotion processing, is a field of further research. Studies measuring possible changes in emotional regulation by continuous positive pressure treatment in patients with sleep apnea would be of highest interest.

## Conclusions

By demonstrating similar emotion processing impairments in patients with two different chronic sleep disorders, our data support the hypothesis of altered emotional regulation as a direct consequence of disturbed sleep

## References

[1] Kret ME, Ploeger A (2015) Emotion processing deficits: a liability spectrum providing insight into comorbidity of mental disorders. Neurosci Biobehav Rev 52: 153–171. S0149-7634(15)00059-7 [pii]; 10.1016/j.neubiorev.2015.02.011 25725415

[2] Baglioni C, Spiegelhalder K, Lombardo C, Riemann D (2010) Sleep and emotions: a focus on insomnia. Sleep Med Rev 14: 227–238. 10.1016/j.smrv.2009.10.007 20137989

[3] Dahl RE (1996) The impact of inadequate sleep on children's daytime cognitive function. Semin Pediatr Neurol 3: 44–50. 8795841 10.1016/s1071-9091(96)80028-3

[4] Genzel L, Spoormaker VI, Konrad BN, Dresler M (2015) The role of rapid eye movement sleep for amygdala-related memory processing. Neurobiol Learn Mem 122: 110–121. S1074-7427(15)00011-8 [pii]; 10.1016/j.nlm.2015.01.008 25638277

[5] Rosales-Lagarde A, Armony JL, Del Rio-Portilla Y, Trejo-Martinez D, Conde R, Corsi-Cabrera M (2012) Enhanced emotional reactivity after selective REM sleep deprivation in humans: an fMRI study. Front Behav Neurosci 6: 25 10.3389/fnbeh.2012.00025 22719723 PMC3376727

[6] Walker MP, van der Helm E (2009) Overnight therapy? The role of sleep in emotional brain processing. Psychol Bull 135: 731–748. 2009-12487-003 [pii]; 10.1037/a0016570 19702380 PMC2890316

[7] van der Helm E, Yao J, Dutt S, Rao V, Saletin JM, Walker MP (2011) REM sleep depotentiates amygdala activity to previous emotional experiences. Curr Biol 21: 2029–2032. S0960-9822(11)01248-6 [pii]; 10.1016/j.cub.2011.10.052 22119526 PMC3237718

[8] Palagini L, Baglioni C, Ciapparelli A, Gemignani A, Riemann D (2013) REM sleep dysregulation in depression: state of the art. Sleep Med Rev 17: 377–390. S1087-0792(12)00120-7 [pii]; 10.1016/j.smrv.2012.11.001 23391633

[9] Riemann D (2009) Does effective management of sleep disorders reduce depressive symptoms and the risk of depression? Drugs 69 Suppl 2: 43–64. 10.2165/11531130-000000000-00000 20047350

[10] Ford DE, Kamerow DB (1989) Epidemiologic study of sleep disturbances and psychiatric disorders. An opportunity for prevention? JAMA 262: 1479–1484. 2769898 10.1001/jama.262.11.1479

[11] Yoo SS, Gujar N, Hu P, Jolesz FA, Walker MP (2007) The human emotional brain without sleep—a prefrontal amygdala disconnect. Curr Biol 17: R877–R878. 17956744 10.1016/j.cub.2007.08.007

[12] Franzen PL, Buysse DJ, Dahl RE, Thompson W, Siegle GJ (2009) Sleep deprivation alters pupillary reactivity to emotional stimuli in healthy young adults. Biol Psychol 80: 300–305. 10.1016/j.biopsycho.2008.10.010 19041689 PMC3107827

[13] Tempesta D, Couyoumdjian A, Curcio G, Moroni F, Marzano C, De GL, et al(2010) Lack of sleep affects the evaluation of emotional stimuli. Brain Res Bull 82: 104–108. 10.1016/j.brainresbull.2010.01.014 20117179

[14] Zohar D, Tzischinsky O, Epstein R, Lavie P (2005) The effects of sleep loss on medical residents' emotional reactions to work events: a cognitive-energy model. Sleep 28: 47–54. 15700720 10.1093/sleep/28.1.47

[15] Anderson C, Platten CR (2011) Sleep deprivation lowers inhibition and enhances impulsivity to negative stimuli. Behav Brain Res 217: 463–466. S0166-4328(10)00656-X [pii]; 10.1016/j.bbr.2010.09.020 20888369

[16] Kessler H, Schwarze M, Filipic S, Traue HC, von WJ (2006) Alexithymia and facial emotion recognition in patients with eating disorders. Int J Eat Disord 39: 245–251. 16485269 10.1002/eat.20228

[17] van der Helm E, Gujar N, Walker MP (2010) Sleep deprivation impairs the accurate recognition of human emotions. Sleep 33: 335–342. 20337191 10.1093/sleep/33.3.335 PMC2831427

[18] Pallesen S, Johnsen BH, Hansen A, Eid J, Thayer JF, Olsen T, Hugdahl K (2004) Sleep deprivation and hemispheric asymmetry for facial recognition reaction time and accuracy. Percept Mot Skills 98: 1305–1314. 15291219 10.2466/pms.98.3c.1305-1314

[19] Ohayon MM (2010) Nocturnal awakenings and difficulty resuming sleep: their burden in the European general population. J Psychosom Res 69: 565–571. 10.1016/j.jpsychores.2010.03.010 21109044

[20] American Academy of Sleep Medicine (2005) ICSD-2—International classification of sleep disorders, 2nd ed.: Diagnostic and coding manual Westchester, Illinois: American Academy of Sleep Medicine.

[21] Kyle SD, Beattie L, Spiegelhalder K, Rogers Z, Espie CA (2014) Altered emotion perception in insomnia disorder. Sleep 37: 775–783. 10.5665/sleep.3588 24899765 PMC4044743

[22] Buysse DJ, Germain A, Hall M, Monk TH, Nofzinger EA (2011) A Neurobiological Model of Insomnia. Drug Discov Today Dis Models 8: 129–137. 10.1016/j.ddmod.2011.07.002 22081772 PMC3212043

[23] Schwartz DJ, Moxley P, Barker A, Longman M (2005) On a characteristic of cortical arousals in individuals with obstructive sleep apnea. J Clin Sleep Med 1: 35–40. 17561613

[24] Cronlein T, Langguth B, Geisler P, Wetter TC, Eichhammer P (2014) Fourteen-day inpatient cognitive-behavioural therapy for insomnia: a logical and useful extension of the stepped-care approach for the treatment of insomnia. Psychother Psychosom 83: 255–256. 000360706 [pii]; 10.1159/000360706 24969136

[25] Buysse DJ, Reynolds CF III, Monk TH, Berman SR, Kupfer DJ (1989) The Pittsburgh Sleep Quality Index: a new instrument for psychiatric practice and research. Psychiatry Res 28: 193–213. 2748771 10.1016/0165-1781(89)90047-4

[26] Kessler H, Bayerl P, Deighton R, Traue H (2002) Facially Expressed Emotion Labeling (FEEL): PC-gestützter Test zur Emotionserkennung. Verhaltenstherapie und Verhaltensmedizin 23: 297–306.

[27] Nofzinger EA, Buysse DJ, Germain A, Price JC, Miewald JM, Kupfer DJ (2004) Functional neuroimaging evidence for hyperarousal in insomnia. Am J Psychiatry 161: 2126–2128. 161/11/2126 [pii]; 10.1176/appi.ajp.161.11.2126 15514418

[28] Beattie L, Kyle SD, Espie CA, Biello SM (2014) Social interactions, emotion and sleep: A systematic review and research agenda. Sleep Med Rev 24C: 83–100. S1087-0792(14)00157-9 [pii]; 10.1016/j.smrv.2014.12.005 25697832

[29] Baglioni C, Spiegelhalder K, Regen W, Feige B, Nissen C, Lombardo C, et al (2014) Insomnia disorder is associated with increased amygdala reactivity to insomnia-related stimuli. Sleep 37: 1907–1917. sp-00562-13 [pii]; 10.5665/sleep.4240 25325493 PMC4548513

[30] Goel N, Rao H, Durmer JS, Dinges DF (2009) Neurocognitive consequences of sleep deprivation. Semin Neurol 29: 320–339. 10.1055/s-0029-1237117 19742409 PMC3564638

[31] Ma N, Dinges DF, Basner M, Rao H (2015) How acute total sleep loss affects the attending brain: a meta-analysis of neuroimaging studies. Sleep 38: 233–240. sp-00284-14 [pii]; 10.5665/sleep.4404 25409102 PMC4288604

[32] Di GM, Martinotti G (2012) Anhedonia and major depression: the role of agomelatine. Eur Neuropsychopharmacol 22 Suppl 3: S505–S510. S0924-977X(12)00185-X [pii]; 10.1016/j.euroneuro.2012.07.004 22959116

[33] Martinotti G, Sepede G, Gambi F, Di IG, De BD, Di NM, et al (2012) Agomelatine versus venlafaxine XR in the treatment of anhedonia in major depressive disorder: a pilot study. J Clin Psychopharmacol 32: 487–491. 10.1097/JCP.0b013e31825d6c25 22722509

[34] Sanchez AI, Martinez P, Miro E, Bardwell WA, Buela-Casal G (2009) CPAP and behavioral therapies in patients with obstructive sleep apnea: effects on daytime sleepiness, mood, and cognitive function. Sleep Med Rev 13: 223–233. 10.1016/j.smrv.2008.07.002 19201228

[35] Ohayon MM (2003) The effects of breathing-related sleep disorders on mood disturbances in the general population. J Clin Psychiatry 64: 1195–1200. 14658968 10.4088/jcp.v64n1009

[36] Sharafkhaneh A, Giray N, Richardson P, Young T, Hirshkowitz M (2005) Association of psychiatric disorders and sleep apnea in a large cohort. Sleep 28: 1405–1411. 16335330 10.1093/sleep/28.11.1405

[37] Ishman SL, Cavey RM, Mettel TL, Gourin CG (2010) Depression, sleepiness, and disease severity in patients with obstructive sleep apnea. Laryngoscope 120: 2331–2335. 10.1002/lary.21111 20939075

[38] Habukawa M, Uchimura N, Kakuma T, Yamamoto K, Ogi K, Hiejima H, et al (2010) Effect of CPAP treatment on residual depressive symptoms in patients with major depression and coexisting sleep apnea: Contribution of daytime sleepiness to residual depressive symptoms. Sleep Med 11: 552–557. 10.1016/j.sleep.2010.02.007 20488748

